# International Consensus Recommendations on the Aesthetic Usage of Ready-to-Use AbobotulinumtoxinA (Alluzience)

**DOI:** 10.1093/asj/sjad222

**Published:** 2023-07-25

**Authors:** Benjamin Ascher, Berthold-Josef Rzany, Philippe Kestemont, Alessio Redaelli, Benoit Hendrickx, Ivano Iozzo, Christoph Martschin, Alicia Milotich, Beatriz Molina, Hugues Cartier, Philippe Picaut, Inna Prygova

## Abstract

Alluzience (abobotulinumtoxinA RTU; Ipsen, Paris, France and Galderma SA, Lausanne, Switzerland) is the first ready-to-use (RTU) botulinum toxin type A liquid solution approved for the treatment of glabellar lines in Europe. In this article, the authors provide consensus recommendations on the aesthetic usage of abobotulinumtoxinA RTU. Members of the International Board on Alluzience convened to develop consensus on the treatment of glabellar lines as well as other facial wrinkles based on their own extensive experience. Consensus recommendations were developed to provide practical guidelines for injection of abobotulinumtoxinA RTU. General guidance on proper assessment, treatment planning, and patient education is provided, as well as specific injection guidelines per indication. Indications covered include glabellar lines, crow's feet, horizontal forehead lines, lateral eyebrow lift, lower eyelid wrinkles, bunny lines, drooping nasal tip, perioral wrinkles, drooping mouth corners, masseter hypertrophy, hollow cheek lines, dimpled chin, and platysmal bands. These guidelines provide a practical framework to support routine injection of facial muscles with Alluzience.

**Level of Evidence: 5:**

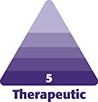

Facial aging is a multifactorial process that involves gravity, muscular facial hyperactivity, soft tissue aging, skeletal remodeling, and skin aging.^1^ With aging, dynamic wrinkles, especially around the central areas, the eyes, forehead, and mouth, eventually become more prominent. Since their introduction more than 30 years ago,^2^ botulinum neurotoxin type A (BoNT-A) injections into the facial muscles have become the most common minimally invasive cosmetic procedure worldwide. In 2021, the International Society of Aesthetic Plastic Surgery (ISAPS) reported over 7 million BoNT-A procedures (up 45% since 2017), with 35- to 50-year-olds accounting for 47.2% of procedures.^3^ When injected into the appropriate facial muscles, BoNT-A inhibits the release of acetylcholine thereby diminishing muscle contraction, and smoothing hyperkinetic wrinkles (eg, mainly glabellar lines, crow's feet, and forehead) and/or correcting facial muscle hyperactivity (eg, gummy smile, masseter etc).

Of the several available preparations of BoNT-A products, ready-to-use (RTU) abobotulinumtoxinA (Alluzience; Ipsen, Paris, France and Galderma SA, Lausanne, Switzerland) was the first RTU liquid solution to be made available in Europe for the treatment of moderate-to-severe glabellar lines.^4^ This RTU liquid formulation was developed to provide several practical benefits over existing lyophilized powder formulations, not only in terms of convenience of injection and prevention of reconstitution errors, but also in terms of consistency and precision of dosing because it is provided at a single RTU concentration that does not require any calculations and is optimized for facial aesthetic use in terms of onset, effect, and duration of effect. The liquid formulation was specifically designed to avoid using any excipients or ingredients from animal or human origin, including lactose and human serum albumin. Indeed, it is the first BoNT-A commercialized product in Europe to successfully remove the need for this human protein, which is used to stabilize other BoNT-A products and prevent adsorption of BoNT-A onto the vial. The aboBoNT-A liquid RTU formulation uses the same purified neurotoxin type A complex (Hall strain) as previous lyophilized powder formulations and contains 0.9% sodium chloride, small amounts of polysorbate 80, histidine to buffer the formulation, and hydrochloric acid to maintain the pH constant at 6.5 (to prevent premature dissociation of the nontoxic neurotoxin-associated proteins and the toxin) (Table 1). Dissociation of the complex is immediate once the product is injected in the targeted tissue.

**Table 1.** The AbobotulinumtoxinA Ready-to-Use Formulation

Ingredient	Function
Clostridium botulinum toxin type A protein complex	Active agent
Histidine	Buffer
Sucrose	Stabilizer
Polysorbate-80	Stabilizer
Sodium chloride	Isotonic agent
Hydrochloric acid	pH adjustment to neutral level
Water for injection	Diluent

In Phase III studies,^5-7^ aboBoNT-A RTU (50 U) was assessed as providing high levels of efficacy in the treatment of moderate-to-severe glabellar lines by both the investigators and patients. With precision dosing, visible effects are seen as early as 24 hours postinjection, with most patients reporting improvement in their wrinkles within 1 to 3 days.^6-8^ A high proportion of patients were considered treatment responders (glabellar line severity rated by investigator as “none” or “mild”) already at Day 8 (80% vs 2.5% with placebo), with peak effect at Day 29, and a significant proportion of patients were still considered as responders at 6 months. The proportion of responders at maximum frown (as determined by the investigator) was maintained over repeated injection cycles (between 82.2% and 87.8%). Importantly for patients, patient satisfaction was very high at peak effect (85.2% vs 9% with placebo) and a significant proportion of patients remained satisfied with their wrinkle appearance at 6 months (49% vs 11% on placebo). Based on the validated FACE-Q patient- reported outcome measure scales for satisfaction with facial appearance overall, significant improvements were observed for psychological well-being and aging appearance.^6-8^ Further investigations of subject satisfaction have been made in the open-label STAR study, which supported high satisfaction through 6 months posttreatment.^9^ As of today, AboBoNT-A RTU is the sole commercialized BoNT-A for which the authorities have granted a 6-month duration effect into the SmPC (Alluzience summary of product characteristics [SmPC]).

In 2010, experts convened an international consensus meeting on the practical use of aboBoNT-A in its powder form (Dysport/Azzalure; Ipsen and Galderma SA) for facial aesthetic indications.^10,11^ Although the core neurotoxin (aboBoNT-A) remains the same, the upgrades in formulation as well as the RTU presentation of Alluzience necessitate specific guidance for the new product that also consider recent important advances in injection technique. The following consensus-led guidelines were developed to provide practical guidance on how to optimize aesthetic outcomes when injecting aboBoNT-A RTU.

## METHODS

The International Board on Alluzience (IBA) comprised 10 dermatologists/plastic surgeons who have extensive experience in the aesthetic usages of aboBoNT-A RTU. Three board members were leading members of the Glabellar Lines Study Group for Alluzience.^6,7^ Board members convened face-to-face on May 14, 2022, to update/develop consensus recommendations on various upper face indications, based on their own experience during clinical practice and based on the results of large clinical studies if available.

Considering the “average” patient, members indicated their preferred injection points on provided anatomic images and discussed the techniques they find useful in clinical practice. High-risk injection points were considered out of scope, as were indications requiring extensive injector experience. Consensus recommendations were developed to provide simple guidelines for the safe injection of aboBoNT-A RTU and reflect the real clinical practice of aesthetic providers. Board members considered aspects such as the recommended injection points, dose, and the correct injection technique. A strong consensus was defined as approval from at least 8 of 10 members. It is important to note that the dosing recommendations provided here refer to aboBoNT-A Speywood Units, which includes Alluzience, as well as the formulations Azzalure and Dysport, and cannot be compared to any other formulations or preparations of BoNT-A.

## GENERAL PREPARATION AND CONSULTATION

The IBA confirmed that the general principles of ensuring proper patient education and counselling from the previous consensus paper^10^ still stand. It remains essential for patients to have a realistic expectation of the treatment outcome as this significantly influences their level of satisfaction with treatment.^12^ Recent surveys have also shown that patients who feel they are well informed and provided with sufficient information are more likely to be satisfied with treatment. Information should be provided in an easy-to-understand manner, avoiding overuse of complicated medical terminology.^13^

During the initial consultation, injectors should discuss in detail the patient's requests and be attentive to the difference between objective and subjective perceptions. Injectors should probe for patient preferences and priorities such as a natural look, or full control of wrinkles, with a risk of fixity. The IBA recommended a static then dynamic approach for mapping and treatment planning. A full holistic assessment of the facial anatomy, during rest and during movement, should be performed and any pre-existing asymmetry documented and discussed. A series of photographs and/or videos with and without muscle movements are crucial before any injection. Injectors may then set out a proposed treatment plan that considers the patient's request, facial anatomy, and skin status while maintaining realistic patient expectations. It is important to consider if the proposed treatment plan will benefit from combining treatments (eg, with dermal fillers or other minimally invasive procedures) and patients should be clearly informed if they are about to receive injections for an off-label indication. Anesthesia is rarely required, and patients should be counselled about the possibility of any expected discomfort as well as the number of injection sites expected for the procedure. Finally, injectors must ensure they remain compliant with all mandatory legal requirements. For example, from October 2021 it is a criminal offence to administer botulinum toxin or a dermal filler by way of injection for a cosmetic purpose to a person aged under 18 in England, even if they have the permission of someone over 18.^14^

In most fields of medicine, shared decision-making is advocated as the preferred model of patient-physician interaction to engage patients in the process of deciding about treatment when more than one reasonable option is available.^15^ Although often not properly implemented in cosmetic medicine,^16^ the IBA considered shared decision-making to be an essential part of the consultation process as it helps determine which products best match the patient's individual characteristics and preferences and forms the basis for true informed consent.^15^ When introducing the aboBoNT-A RTU liquid formulation to patients, the IBA considered that it is important to mention to the patients that the upgraded RTU formulation contains the same, proven, well-studied neurotoxin as the powder formulations (Azzalure/Dysport). This enables a thorough discussion of the proven mode of action, long history of usage, and good safety record of aboBoNT-A. In line with patient expectations for an aesthetic product, the innovative formulation has also enabled a switch to plant-based and synthetic excipients. Some patients may also be reassured that the formulation makes it possible to increase the precision of the dosing by not going through a reconstitution phase and uses novel syringes with the units and volume already calibrated. In the recent open-label STAR study, investigators reported multiple benefits with the RTU product, including precision of the injection and the possibility to spend more time on patients.^9^

Patients should be made aware of the estimated onset of action and duration of effect of aboBoNT-A RTU. Of note, the large pivotal studies conducted to gain approval in Europe support a distinctly favorable time course of action. Up to a quarter of patients experience improvements within the first day of injection, and the median onset was within 3 days. AboBoNT-A RTU has also demonstrated a long duration of action of up to 6 months postinjection and with maintained efficacy over repeated cycles.^7^ Injectors should also explain how the treatment will be delivered (eg, number of injection points), potential adverse events (eg, headache and/or injection site reactions^9,17^), and, if necessary, the treatment to correct any undesired outcomes. While some injection site pain can be expected, it is typically transient (gone within seconds) and was reported as an adverse event in only 1% to 5% of patients in the clinical trials.^5,7^ In the STAR study, 91% of surveyed patients said they “felt comfortable with the injection procedure.”^9^ Patients should also be counseled that because the effects of aboBoNT-A RTU are potentially longer lasting than those of other BoNT-A products, there needs to be full agreement on their treatment goals and expectations. A follow-up appointment may be scheduled, the benefits of which have been shown to be another significant contributor to patient satisfaction.^13^ All patients should provide documented informed consent before treatment commences.

## CONSENSUS GUIDELINES

### Glabellar Lines

**Figure 1.**
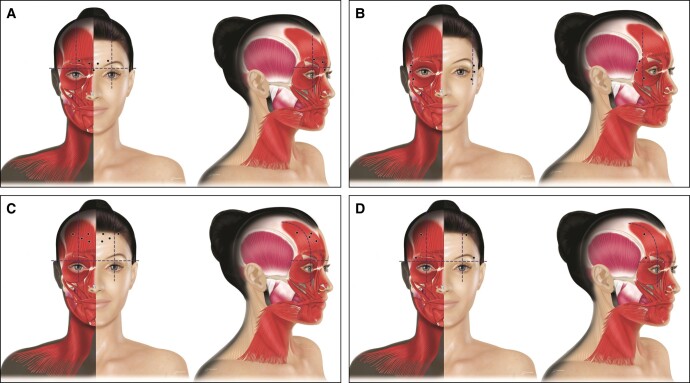
Treatment of (A) glabellar lines, (B) crow’s feet, (C) horizontal forehead lines, and (D) lateral brow lift. For the treatment of glabellar lines, a 5-point injection is recommended for most patients, with 1 point in the procerus and 2 points in each corrugator. For the treatment of crow's feet, an injection pattern of 3 points per side (ie, 6 points in total) surrounding the canthal region is recommended for most patients. For the treatment of horizontal forehead lines, the International Board on Alluzience recommended 4 to 8 points for the total frontalis depending on patient presentation (gigure shows 8 points). For the lateral brow lift, 1 injection point should be placed at each eyebrow tail into the pars orbicularis, 1 cm above the orbital rim. An additional injection point should be placed at the external part of the frontalis, at a minimum of 4.5 cm up from the eyebrow (depending on hairline) and 4 to 5 cm from the midline. Created by and published with permission from Virginie Denis.

AboBoNT-A RTU is indicated for the improvement in appearance of moderate to severe glabellar lines (vertical lines between the eyebrows) and its efficacy and safety has been established in recent randomized, controlled studies.^5,6,8^ For the treatment of glabellar lines, as per the labelling guidelines, a 5-point injection is recommended for most patients, with 1 point in the procerus and 2 points in each corrugator (Figure 1A). In the case of a long and/or strong procerus, the IBA recommended that a second injection point along the same procerus crease can be made. In the case of long corrugators (extending beyond the midpupillary line) a third injection point per corrugator can be added laterally.

Injections should be perpendicular (keeping the distance between skin and target as short as possible to encounter fewer blood vessels and free nerve endings, thereby inducing less bruising and pain), 0.5 to 1 cm from the upper orbital rims and internal to the midpupillary lines and never in the direction of the orbital rim. IBA members recommended injecting deep medially and more superficial laterally. The recommended total aboBoNT-A RTU dose is 50 U, with 10 U in the procerus, 10 U in the head of each corrugator, and 10 U in the tail of each corrugator. If a third injection per corrugator is required, IBA members recommended administering 2.5 U lateral to the midpupillary line (intramuscularly or subdermally depending upon muscle size). Some of the IBA board members preferred to consider this “extra” injection at a touch-up visit conducted 2 to 4 weeks after the initial injection. There was consensus that the final dosage for each individual patient depends on their muscle structure and mass, the wrinkle severity, and patient's preference of a more natural or a more static look.

IBA members noted that the need for procerus injection may decrease over time, but 70% of members said they continue to inject because it elevates the medial part of the brow. Some advisors noted they preferred to inject within skin pores because this helps avoid nerves and blood vessels. As with prior formulations, injection site reactions, including transient injection site pain (typically lasting a few seconds) and hematoma, remain the main safety concerns for this indication.^10,11^ Injection site reactions can mostly be mitigated with proper injection technique as can eyelid ptosis (caused by unwanted involvement of the levator palpebrae).^18^ Adverse events such as headache or eyelid ptosis rarely occur and usually subside within days or a few weeks.^7^ Patients with a history of eyelid ptosis following treatment should be counseled that their individual anatomy might be predisposed to the diffusion of toxin into the levator palpebrae superioris.^19^

### Dynamic and Static Periorbital Wrinkles (Crow's Feet)

Lateral canthal rhytids (crow's feet) are a common indication where injections are used to compensate for overactivation of orbicularis oculi during animation. The injection technique may also be part of a nonsurgical brow lift. For the treatment of crow's feet, the IBA recommended injecting 3 points per side (ie, 6 points in total) surrounding the canthal region (Figure 1B). For some patients with long and severe crow's feet the number of injections can be increased to 6 points per side adapted to the location of the wrinkles. All points should be at the lateral part of the orbicularis oculi, 1 to 2 cm from the external orbital rim. Some board members recommended that the inferior point should be a bit more internal and/or a lower dose to avoid any diffusion to the zygomatic muscles. Injections should be slow and controlled, lateral (30-45° angle to the skin), and superficially into the dermal plane. It is recommended that the needle always points away from the patient's eyes. The injector should stretch the skin slightly to visualize superficial veins and avoid bruising. A total aboBoNT-A RTU dose of 30 to 60 U is recommended, with 5 to 10 U per point (15-30 U per side); dosage considerations include the strength of the muscle, depth of wrinkles, and the thickness/firmness of the skin and subcutaneous tissue. Doses should be reduced (mainly in the inferior part) for patients with signs of malar edema. As with previous aboBoNT-A formulations, the minimal injection dose should be adopted to avoid a frozen look when smiling.

Unwanted effects include pain, aggravation of malar edema and under-eye wrinkles, and effects on neighboring muscles such as the inferior region of the zygomaticus major, which can lead to drooping mouth corners and asymmetry if injected. To avoid these problems, the IBA recommended performing a snap test to confirm the patient has adequate skin elasticity and to avoid injecting the canthal region in patients with prominent malar edema. Treatment of crow's feet can be combined with lower eyelid wrinkles if they are present. In this case, the same injection points should be used with a slightly lower dose per point. Eyelid ptosis is avoided by injecting superficially with posterior directionality.

### Horizontal Forehead Lines

Often treated alongside the glabellar region, horizontal forehead lines are another commonly treated indication for BoNT-A injections. For the treatment of horizontal forehead lines, the IBA recommended 4 to 8 points for the total frontalis depending on patient presentation. In view of the anatomy of the frontalis muscles and thin skin of this location the injections should be superficial (ie, subdermal/intramuscular), and perpendicular to the skin. To avoid any brow ptosis, the IBA recommended injecting relatively high on the forehead (at least 2 cm from the upper orbital rim and avoiding the inferior frontalis), in a slightly curved V-shape for women and more horizontally in men (Figure 1C). A total aboBoNT-A RTU dose range of 20 to 40 U (2.5-5 U per point) was recommended. The higher the number of injection points, the lower the dose per point should be used.

IBA members also recommended an injection (2.5 U) into the orbicularis point, 1 cm above the orbital rim, to protect the effect. Half of the IBA board members recommended starting with a smaller dose followed by a touch-up visit, while the other half only performed a touch up if needed. Board members noted that movement of the lateral frontalis can sometimes lead to fine wrinkles (WIFI lines) above the lateral part of the eyebrows. This can be treated with a subdermal/intradermal injection of 2.5 U in the WIFI line, during the full injection or during the touch up.

### Lateral Eyebrow Lift

Usually performed in women and in combination with other upper face treatments, BoNT-A treatment is often used to correct dropping of the lateral part of the brow. For this indication, the IBA recommended a 4-point injection with 2 points per side (Figure 1D). One injection point should be placed at each eyebrow tail into the pars orbitalis, 1 cm above the orbital rim. Treatment with aboBoNT-A RTU blocks the depressor while the frontalis functions normally to elevate the lateral part of the eyebrow. An additional injection point should be placed at the external part of the frontalis, at a minimum of 4.5 cm and an average of 4 cm from the midline. Injection in this part of the frontalis boosts the levator activity of the inferior part of the frontalis, and can further drop the medial brow, reshape the entire eyebrow, and accentuate the lateral arch.

The recommended dose is 5 to 10 U/point and the total dose range is 20 to 40 U. Injection into the pars orbicularis should be of medium depth (6 mm) and the injection into the frontalis should superficial, intramuscular, and perpendicular to the skin. Eyelid and brow ptosis occur only rarely when the injection sites are too close to the orbital rim.

### Lower Eyelid Wrinkles

**Figure 2.**
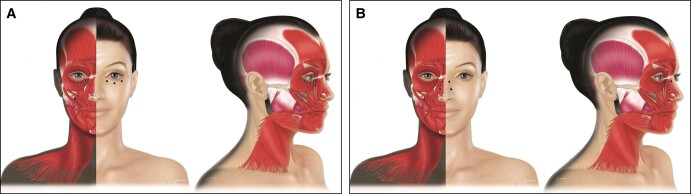
Treatment of (A) lower eyelid wrinkles and (B) bunny lines. For the treatment of lower eyelid wrinkles, board members recommended 1 to 3 injections about 2 to 3 mm below the lash line. For the treatment of bunny lines, 1 to 3 injection points in the nasalis pars transversa are recommended (figure shows 2 points per side). Created by and published with permission from Virginie Denis.

Lower eyelid wrinkles (external and inferior eyelid orbicularis oculi bands) occur because of overactivity of the tarsal and lacrimal portions of orbicularis oculi. For the treatment of lower eyelid wrinkles, board members recommended 1 to 3 injections about 2 to 3 mm below the lash line. Injections can be made at the sand, “owwee,” and midpupillary points^20^ (Figure 2A). Board members recommended injecting tangentially and very superficially (with papule) 1 to 2 U per point for a total dose of 5 to 6 U (2.5-3 U per side). Treatment of lower eyelid wrinkles may be synergistic with treatment of the lateral periorbital wrinkles (“crow's feet”) to obtain optimal results. IBA members agreed that treatment is not suitable for treating the static wrinkles caused by photodamage or eye bags caused by muscle laxity; in these cases, combination therapy with fillers, peeling, lasers, or surgery is usually more appropriate.

Unwanted effects for both indications include ectropion and exacerbating dry eye. Injectors should ensure patients have sufficient lid elasticity using a snap test (<2 seconds), and should avoid injecting patients with dry eyes, prominent malar edema, scleral show, or morning eyelid edema.

### Open Eye Technique

Treatment with BoNT-A reduces the inferior wrinkles, increases the palpebral aperture, and thus widens the eyes. The open eye technique is used where there are asymmetries of the eyelids or to treat narrow eyes on smiling and facial palsy. For this technique, the IBA recommended 2 to 3 superficial injection points (1-2 U per point for a total dose of 4-6 U) on the lower lid, close to the lash line in the lateral and tarsal parts of the orbicularis oculi. In some cases, when the patient has small eyes or wants to elevate their eyebrows, an additional 3 injections in the lateral canthus can help improve symmetry.

As with lower eyelid wrinkles, unwanted effects include ectropion and exacerbating dry eye. Patient exclusion criteria are like those for crow's feet. Injectors should ensure patients have sufficient skin elasticity using a snap test (<2 seconds), and should avoid injecting patients with dry eyes, prominent malar edema, scleral show, or morning eyelid edema.

### Bunny Lines

Bunny lines refer to dynamic wrinkles (from the levator labii superioris alaeque nasi and the transverse nasalis muscles, together with the medial portion of the orbicularis oculi muscle) on the side of the nose. In some patients, dynamic wrinkles may extend to the lower eyelids and cheeks. If bunny lines appear in addition to glabellar lines when patients frown, they should be treated together. Weakening of the lateral portion of the orbicularis oculi muscle for reduction of lateral canthal lines often results in compensatory hyperactivity of the medial portion, accentuating the bunny lines. For the treatment of bunny lines, 1 to 3 injection points in the nasalis pars transversa are recommended (Figure 2B). In case of wider lines, additional injections into the alae nasi part of the levator labii alaeque nasi (LLAN) are also recommended. Injections should be deep (close to the periosteum), the orientation of the injection should be perpendicular, with an angle of about 90° to the nasal bone. The consensus recommendation is to use aboBoNT-A RTU 2.5 to 10 U per injection point for a total dose of 10 to 20 U.

### Injecting for the Upper Face and Overall Facial Enhancement

Treatment planning should consider whether the patient's goals and preferences are for one specific area or for overall facial shaping and enhancement. Facial muscles do not act in isolation but have complex interactions, and the treatment plan must consider the interdigitation of muscle fibers and the degree to which their activities are antagonistic to each other to ensure the desired aesthetic effect is achieved. For example, the most common indications of glabellar, lateral canthal, or forehead lines can all alter eyebrow shape and position, and this should be considered when planning and discussing treatment plans with the patient.^21,22^ In addition, facial muscles with different functions and orientations often overlap and cross various planes, making the depth of injection an important consideration. Injections made too superficial or too deep can lead to inadvertent injection of the wrong muscle, potentially causing an opposite effect from the desired outcome.^23^ Finally, facial asymmetry can occur if injections are administered unequally between both sides of the face.

### Drooping Nasal Tip

A drooping nasal tip when smiling often involves increased activity of the depressor septi nasi or hypertonia of the LLAN. To prevent the nasal tip from drooping with animation, 1 injection in the middle of the columella is recommended. The aboBoNT-A RTU dose should be 10 U, and the injection should be perpendicular to the skin and deep in the region of the nasal spine, as both muscle bellies cross the midline at this point. If there is hypertonia of the LLAN muscle with a clear lift of the nasal sides and rotation of the tip downwards, some IBA members recommended injecting an additional point on each side at the sides of the alae nasi part of the LLAN. In this case, the aboBoNT-A RTU dose should be 10 U per point.

Pain is the most common adverse event and topical numbing creams are recommended. Patients should not be treated for nasal tip if the nose does not droop when smiling and talking. Injections into the LLAN should be made with caution because it is possible that the length of the upper lip can increase, resulting in ptosis. Other indications in the nasal area (eg, in the alar nasalis to decrease the size of nostril aperture) are only recommended for experienced injectors.

### Perioral Wrinkles

**Figure 3.**
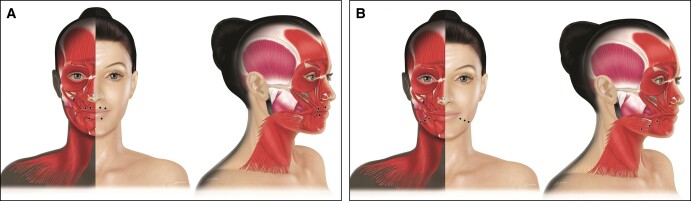
Treatment of (A) perioral wrinkles and (B) drooping mouth corners. For the treatment of perioral wrinkles, 4 to 6 injection points are recommended, with 4 symmetric points just above the vermilion border and, if necessary, 2 points on the lower lip. For the treatment of drooping mouth corners, a 1- to 2-point injection into the depressor anguli oris is recommended. The major injection points should be 1 cm lateral to the commissure and 1 cm down. If additional hyperkinetic wrinkles appear lateral to the lip corner, the International Board on Alluzience recommended also injecting the fascial platysma (inferior point). Created by and published with permission from Virginie Denis.

The perioral area is a complex area due to its function and the interplay of muscles. Aging, heliodermy, and smoking can result in substantial changes in the appearance of the lips including vertical perioral wrinkles. The normal actions of the orbicularis oris accentuate these lines. For the treatment of perioral wrinkles, 4 to 6 injection points are recommended, with four symmetric points just above the vermilion border and, if necessary, 2 points on the lower lip (Figure 3A). Injections should be perpendicular to the skin and intradermal, and injection sites should be adjusted to the pattern of perioral lines. The IBA advised that lateral points should be at least 1.5 cm away from the mouth corners to avoid incompetence, which may cause drooling as well as difficulties in speaking, drinking, and eating.

A total dose of 4 to 12 U is recommended, with 1 to 2 U per point. The dose depends on the muscle strength, severity of the hyperkinetic lines, and the degree of elastosis. However, higher doses should be avoided in the cupid’s bow. Due to the complexity of the area, the IBA recommended a conservative approach to treatment because overcorrection can cause significant mouth dysfunction. A reasonable approach is to begin with 1 site per quarter lip and reassess in 2 weeks when a touch up or additional injection sites can be added. Combination treatment with dermal fillers is often indicated in older patients to preserve the shape of the philtrum. Injections may be painful, and application of a numbing cream is recommended. Finally, patients should be counseled that complete wrinkle removal is not realistic and that combined therapy with dermal fillers, laser, peelings, and/or energy-based devices can be necessary. Even in the best hands, patients should be prepared to expect some difficulty of speech, drinking with a straw, or excessive pouting of the lips in the first few days. It is therefore recommended to start with low doses and to gradually increase the dose upon repeated treatments.

### Drooping Mouth Corners

Drooping mouth corners can make a face appear sad or angry and are also commonly associated with marionette lines that run down from the corners of the mouth. A 1- to 2-point injection into the depressor anguli oris is recommended (Figure 3B). The major injection points should be 1 cm lateral to the commissure and 1 cm down. The injector should pinch the muscle slightly to prevent its movement and inject intramuscularly. The needle should point towards the side with a posterior flow to avoid the depressor labi inferioris which could cause labial incompetence. A total dose of 5 to 10 U is recommended, with 2.5 to 5 U per point depending on the strength of the depressor. If additional hyperkinetic wrinkles appear lateral to the lip corner, the IBA recommended also injecting the fascial platysma using an additional 2 injection points (5 U per point).

For this indication, the IBA noted that injections will not improve severely depressed oral commissures and will not elevate the marionette lines. It is therefore important to inform the patient that only a subtle improvement is likely. For these patients, a combination strategy with aboBoNT-A RTU and a dermal filler can augment the quality of the result.

### Masseter Hypertrophy and Hollow Cheek Lines

The masseter is a large, strong muscle in an active area of the face. AboBoNT-A RTU injections can be used to temporarily weaken the masseter, resulting in a smoother and slimmer lower face contour. This procedure is common mainly in Asian patients and good results can be achieved with repeated injections. In Caucasians, masseter hypertrophy is more often associated with bruxism, which also responds well to treatment by toxin following an adapted protocol.

**Figure 4.**
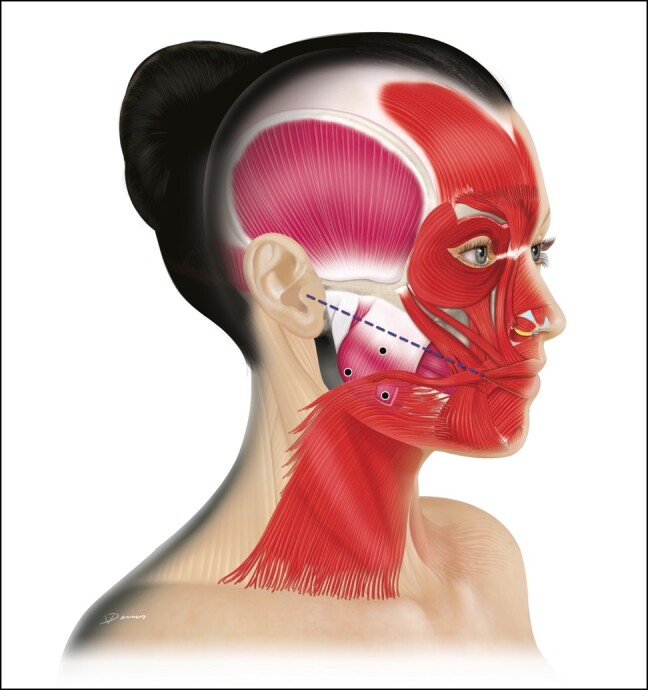
Treatment of masseter hypertrophy. For masseter hypertrophy, a pyramid of 3 to 5 injection points per side is recommended (figure shows 3 points per side). Created by and published with permission from Virginie Denis.

For masseter hypertrophy, the IBA recommended 3 to 5 injection points (in a pyramid) per side (Figure 4). Injections should be perpendicular, injecting first deep and then superficially into the 2 portions of the muscle. The total dose should be 40 to 80 U (5-10 U per point) per site depending on masseter strength. Higher doses of aboBoNT-A (100-140 U) have been reported in Asian patients.^24^ Injections should be avoided above the line from the lip corner to tragus. Injection just beneath the zygomatic bone should be avoided to prevent unwanted effects in the zygomatic muscles which can lead to awkward facial expression, especially when smiling. Due to possible effects on crunching power and mastication capability, it is important to assess the muscle mass and adjust doses accordingly. Finally, the IBA noted that the temporalis works in synergy with the masseter, and in some cases of bruxism, some IBA members prefer to simultaneously inject the upper temporalis (above the hairline) using an additional 4 injection points (5 U per point) close to the temporal crest. This is not required for facial contouring unless there is visible hypertrophy of the temporalis muscle.

**Figure 5.**
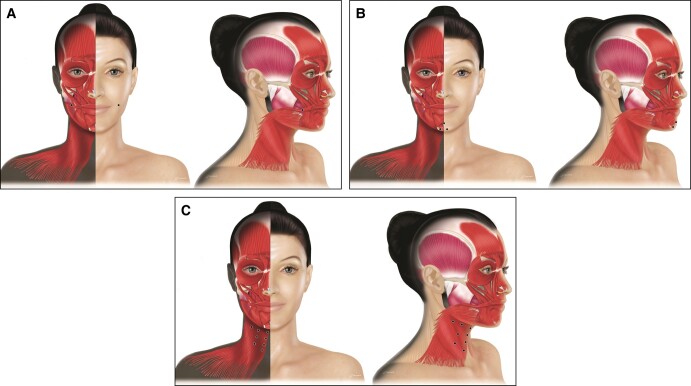
Treatment of (A) hollow cheek lines, (B) dimpled chin, and (C) platysmal bands. For the treatment of hollow cheek lines, the International Board on Alluzience recommended injecting 1 to 2 points per side, at least 1 cm behind the modiolus. Injections should be intradermal and have a posterior flow following the wrinkle. To treat the dimpled chin, a 1- to 3-point injection at the bony jawline close to the central midline is recommended. For platysmal bands, it is recommended to start the first point at the jawline and go down every 2 cm to the middle of the bands. Created by and published with permission from Virginie Denis.

Hollow lines of the cheek are a relatively rare indication caused by hypertonus of the risorius. The IBA recommended injecting 1 to 2 points per side, at least 1 cm behind the modiolus (Figure 5A). Injections should be intradermal and have a posterior flow following the wrinkle, and the recommended aboBoNT-A dose is 1 to 2 U per point. Treatment is usually combined with a dermal filler. Injections should be superficial to avoid the buccinator muscle, and injectors should be careful to address any asymmetries.

### Dimpled Chin

A dimpled chin is caused by contraction of the mentalis muscle, and the IBA noted they prefer to inject only patients with active dimpling (eg, they dimple while speaking). Combination therapy with fillers is often appropriate because loss of collagen and subcutaneous fat in this region is a significant contributor to the formation of a dimpled chin.

The IBA recommended a 1- to 3-point injection at the bony jaw line close to the central midline (Figure 5B). Injections should be deep with an aboBoNT-A RTU dose of 5 to 10 U per injection point adjusted according to the mentalis muscle mass. Injecting laterally and at a higher-than-recommended dose may affect the depressor labii inferioris, causing mouth asymmetry. Injections can be supplemented with some subdermal injections into the dimples as required. The IBA noted that when the dimpled chin and drooping mouth corners (depressor anguli oris) are treated together, 1 central injection point of 5 to 10 U might be sufficient. Safety concerns mainly relate to using higher-than-recommended doses which may affect the depressor labii inferioris and the orbicularis oris, causing drooling, speech impairment, and mouth asymmetry.

### Platysmal Bands and Décolleté Wrinkles

Visible active platysmal bands on the neck may be apparent in some slim patients and become more prominent when they speak or smile. Patients should be seated upright, and the injector should pinch each band and inject laterally with a superficially intramuscular injection. As with all aesthetic indications, there is usually no need for topical anesthesia.

The total number of injection points depends on the number and length of platysmal bands; it is recommended to start the first point at the jawline and go down every 2 cm to the middle of the bands (Figure 5C). The total maximum dose recommended for this indication is typically 100 U, with a total of 20 U per treated band (5-10 U every 2 cm depending on neck length). Injections can be perpendicular to lateral in an external direction. There is no need to inject at the midline because there is no muscle in this region and there is a risk of spread to the subhyoid muscle which can cause significant problems with breathing or swallowing. The posterior part of the platysma should be avoided in high doses due to the risk of toxin spread to the sternocleidomastoid muscle with the risk of causing a posterior tilt of the head. The IBA recommended scheduling a follow-up visit at 2 weeks for touch up or treatment if new smaller bands appear.

The IBA did not typically recommend treatment with BoNT-A for the décolleté region where aging is usually caused by photodamage. Instead, they preferred alternatives such as mesotherapy, lipofilling, dermal fillers, and/or lasers, which are often more effective. When used (in rare cases) the IBA recommended a maximum total dose of 120 U with 2.5 to 5 U per point. Injections should follow the distribution of wrinkling in the pectoralis muscle. When necessary, platysmal bands should be treated together with the décolleté wrinkles.

## SUMMARY AND ADDITIONAL POINTS FOR CONSIDERATION

These guidelines provide a practical framework for the routine injection of facial muscles with aboBoNT-A RTU (Alluzience). The guidelines were prepared to address the initial approach for each indication based on the IBA's extensive experience with the product and its characteristics and knowledge on anatomy and physiology. A thorough understanding of static and dynamic facial anatomy is a prerequisite for anyone undertaking aesthetic procedures with aboBoNT-A RTU to maximize efficacy, and minimize the risk of adverse events. Board members considered thorough assessment and a proper discussion of treatment objectives as fundamental to treatment success. This is because doses and injection techniques (eg, depth of injection) should be optimized to the individual patient, considering their unique facial anatomy, and based on the size and shape of the muscle, the severity of the wrinkles, and the degree of immobilization required by the patient.

The IBA recommends that injectors should always aim to start with the lowest recommended effective dose per muscle for the first injection, and to work in an iterative manner; some patients will benefit from a touch-up visit 2 to 4 weeks after the initial injection. Using lower-than-recommended doses may result in suboptimal efficacy or duration of effect. Once the patient has achieved their goal, the injector will need to consider maintenance treatment, taking into consideration that, although the efficacy of injection does not change with repeat treatment, the original muscle balance has been modified. The minimum injection interval is 3 months; however, the efficiency of the Alluzience formulation typically allows for a longer duration of benefit. Other maintenance considerations include aspects such as stabilization of the eyebrow position and the prevention of new wrinkles appearing (eg, injection into new faint lines). For patients with significant volume loss due to aging, injectors can consider combination treatments to correct for volume loss.^25,26^ For younger patients whose goals are preventative, the IBA recommended a similar treatment approach considering whether patients can use a slightly lower dose or have a longer interval between treatments. As for all patients, there is a duty for injectors to ensure that the younger patient has a good understanding of what can be achieved with such treatment.
